# Multimodal Wrist Biosensor for Wearable Cuff-less Blood Pressure Monitoring System

**DOI:** 10.1038/s41598-019-44348-3

**Published:** 2019-05-28

**Authors:** Vega Pradana Rachim, Wan-Young Chung

**Affiliations:** Department of Electronic Engineering, Pukyong National University, Busan, 48513 South Korea

**Keywords:** Information technology, Electrical and electronic engineering, Biomedical engineering, Hypertension

## Abstract

We propose a multimodal biosensor for use in continuous blood pressure (BP) monitoring system. Our proposed novel configuration measures photo-plethysmography (PPG) and impedance plethysmography (IPG) signals simultaneously from the subject wrist. The proposed biosensor system enables a fully non-intrusive system that is cuff-less, also utilize a single measurement site for maximum wearability and convenience of the patients. The efficacy of the proposed technique was evaluated on 10 young healthy subjects. Experimental results indicate that the pulse transit time (PTT)-based features calculated from an IPG peak and PPG maximum second derivative (f_14_) had a relatively high correlation coefficient (*r*) to the reference BP, with −0.81 ± 0.08 and −0.78 ± 0.09 for systolic BP (SBP) and diastolic BP (DBP), respectively. Moreover, here we proposed two BP estimation models that utilize six- and one-point calibration models. The six-point model is based on the PTT, whereas the one-point model is based on the combined PTT and radial impedance (Z). Thus, in both models, we observed an adequate root-mean-square-error estimation performance, with 4.20 ± 1.66 and 2.90 ± 0.90 for SBP and DBP, respectively, with the PTT BP model; and 6.86 ± 1.65 and 6.67 ± 1.75 for SBP and DBP, respectively, with the PTT-Z BP model. This study suggests the possibility of estimating a subject’s BP from only wrist bio-signals. Thus, the six- and one-point PTT-Z calibration models offer adequate performance for practical applications.

## Introduction

Hypertension is a condition in which the force of blood flow through the blood vessel consistently fluctuates outside the desired normal ranges. According to the World Health Organization, hypertension is estimated to cause 7.5 million deaths, that is, 12.8% of total deaths worldwide^1^. Moreover, elevated blood pressure (BP) is a major risk factor for cardiovascular diseases (CVD) and chronic kidney disease (CKD) as well as hemorrhagic stroke^2^. Recent studies in the US and South Korea reveals that young adults subject (18 to 39 years of age) that suffered from hypertension are at significantly increased risk of CVD and stroke later in their life^3,4^. As no obvious symptoms of elevated BP can be observed, preventative actions based on healthy lifestyle choices represent the only means of controlling BP and thus ensuring healthier and longer lives. Therefore, the development of continuous home BP and 24-hour ambulatory BP monitoring is emerging and critical to ensuring proper healthcare treatment and management.

The conventional technique for non-invasive blood pressure measurement is using a sphygmomanometer with an inflatable cuff. However, this is used only for discrete measurements. More importantly, it is obstructive and requires that medical experts perform an auscultation technique in which a diaphragm stethoscope is placed over the brachial artery to listen to the Korotkoff sound while the pressure is controlled under the cuff. Thus, a cuff-less BP technology that offers continuous, highly wearable, and non-intrusive measurement must be developed. Several cuff-less methods have been successfully developed by researchers mainly based on pulse transit time (PTT)^5^ and pulse wave analysis (PWA). Recently, Yoon *et al*.^6^ showed the possibilities of estimating BP values using a PWA-BP multi-regression model. The most common method utilizes electrocardiogram (ECG) and photo-plethysmography (PPG) signals to calculate pulse arrival time (PAT)^7,8^. However, Martin *et al*.^9^ compared the conventional PAT-based BP model with their BCG scale-PTT-based BP model and, after multiple BP interventions experiment, they concluded that the PAT-based model is effective only with systolic BP (SBP) and not diastolic BP (DBP) measurements^8,10^.

In this study, we propose a multimodal wrist biosensor in which both IPG and PPG sensors are attached to the back of a wristband strap to enable continuous, yet cuff-less blood pressure (BP) monitoring system. In this stage of research, we limited our target population into the young healthy adults as it is shown that the largest increases in hypertension prevalence have occurred in the young adult population^11^. Thus, the proposed system is targeted for the initial BP screening and early detection of elevated BP and hypertension. Our proposed configuration tackles the common complicated setup using the PTT BP estimation method that requires multiple sensor placements on the subject’s chest, foot, or finger^7–9^, by proposing a compact non-intrusive single site wrist measurement. Furthermore, no consensus has been reached on an exact PTT definition. Thus, in this study, we define 14 PTT-features as f_1_–f_14_ and identify the best PTT feature that correlates well with both SBP and DBP and that is suitable for the PTT BP estimation model. Moreover, we compare the PTT value from the multimodal biosensor with the conventional PAT value from the subject’s chest ECG and finger PPG. We also introduce an additional feature defined as radial impedance (Z) for the one-point BP calibration model (also known as the PTT-Z BP estimation model). Finally, the results of designed arm-exercise intervention experiments with the PTT BP and PTT-Z BP models are compared for all subjects.

## Methods

### Data collection and system design

In total, 10 young healthy volunteers (25 ± 4 years; gender: 6 males and 4 females; weight: 63 ± 8; height: 165 ± 6 cm) with no histories of hypertension or cardiovascular disease were enrolled in our study. Written informed consent was obtained from participants after we had provided them with a complete description of the study. This study was approved by the ethics committee of Pukyong National University and conducted according to the Declaration of Helsinki ethical principles for medical research on human subjects.

**Figure 1:**
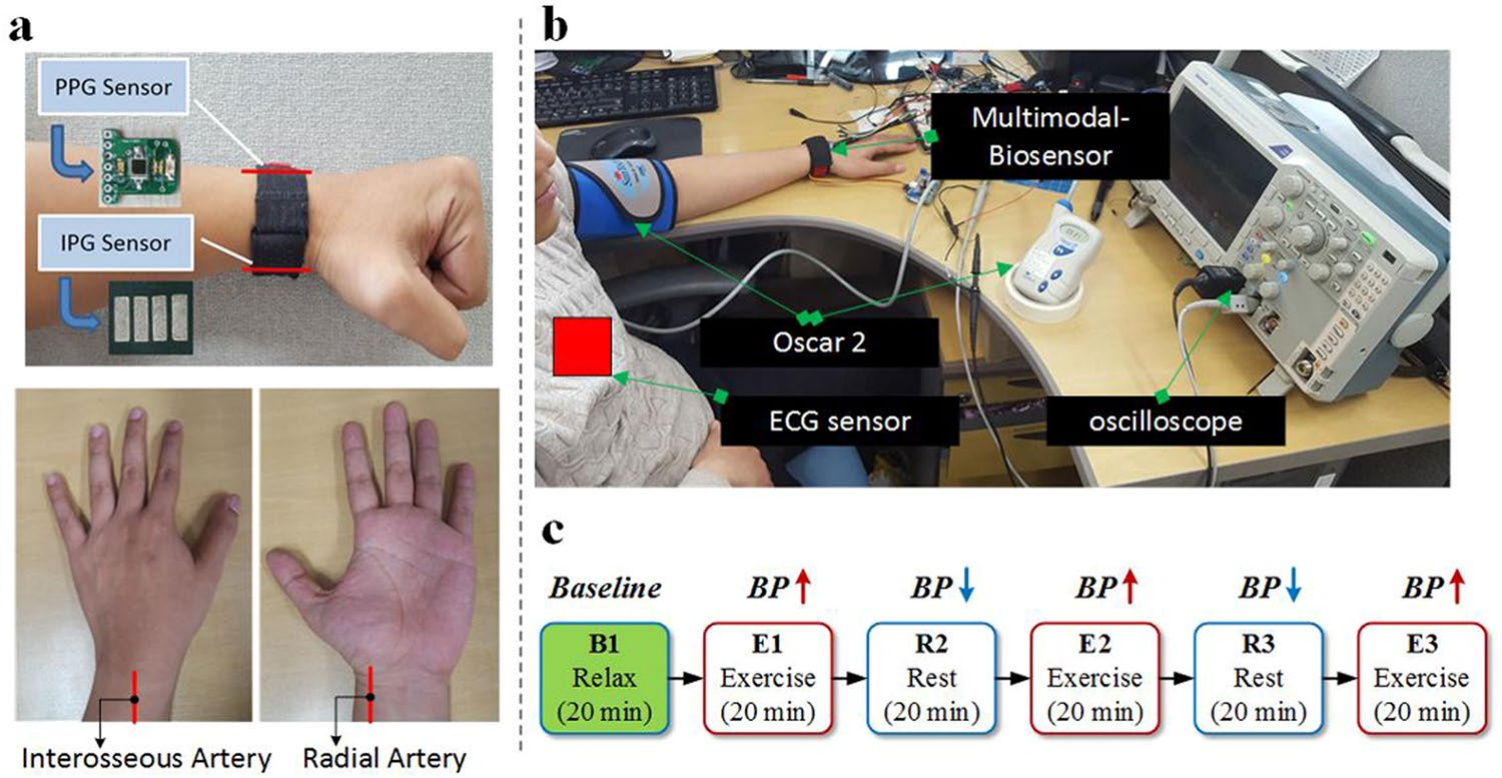
(**a**) Multimodal biosensor with an impedance plethysmography (IPG) signal acted as a proximal waveform, and a photo plethysmography (PPG) signal acted as a distal waveform. (**b**) Measurement setup with chest electrocardiogram (ECG), wrist PPG, and wrist IPG collected simultaneously, and left-arm blood pressure (BP) monitoring with Oscar 2 for BP reference data acquisition. (**c**) Measurement protocol with three rest sessions (R1, R2, R3) and three arm-exercise sessions (E1, E2, E3) to increase BP.

In each experiment, the subject followed the same measurement setup and protocol as shown in Fig. 1. Four physiological waveforms were measured simultaneously during the experiments. Our proposed multimodal biosensor was worn on the left wrist of each subject. First, we used the IPG sensor to measure the radial impedance of the radial artery from the inner part of the subject wrist. Second, we employed the PPG sensor for the arterial blood volume pulsation measurement in the area of interosseous arteries from the outer part of the subject wrist (Fig. 1a). Third, an ECG sensor located on the subject chest was used to record the electrical activity of the heart of each subject, which acted as the proximal waveform in the PAT calculation. Lastly, we utilized Oscar 2 (SunTech Medical, USA) for BP reference data acquisition (Fig. 1b). This device is considered the gold standard in 24-hour ambulatory BP monitoring and is certified by the British Hypertension Society (BHS) and the AAMI-SP10 standards.

The details of the proposed multimodal biosensors system are given as follow. The proposed prototype device is attached to the back of the wrist strap and directly contacts the subject’s skin. For the PPG sensor, the overall design was described in our previous report^12^. In brief, we use four LEDs to transmit light in different optical wavelengths (in both visible and near-infrared optical spectra) ranging between 660–940 nm. However, only the PPG signal collected from 940 nm is used for analysis, multi-channel PPG signals can be used for heart rate (HR) monitoring during intensive exercise, the motion artifacts reduction algorithm from multi-channel PPG signal can be utilized to separate HR signal and the motion artifacts in the time domain. Moreover, the remaining signal is intended for another clinical experiment such as peripheral capillary oxygen saturation monitoring^13^.

The size of the prototype PPG sensor is 15 × 15 mm^2^ and we utilize a photodiode (PD) from BPW 34 (OSRAM Semiconductor Inc.) to detect reflected light from the subject wrist skin. Moreover, the proposed IPG sensor consists of four conductive silver-plated polyester electrodes that are flexible, stretchable in both directions, and suitable for long-term healthcare monitoring^14^. Each electrode is sewed to the strap and acts as a voltage detector and current injector. In this study, we used 500 *μA* from a 100-kHz current source, which is considered safe for the human body and sufficiently assists in detecting the impedance variance^15^. In addition, a three-channel wet electrode with an AD8232 (Analog Devices, Inc., USA) single-lead front end was used for ECG signal collection.

Our experiments were performed repeatedly in an indoor laboratory from April to June 2018 under an ambient temperature controlled at 25 °C and a relative humidity of 60%. For data collection with each subject, a recorded signal was started approximately 5 s before the cuffed BP measurement was taken using Oscar 2 and finished approximately 1 min after the cuff was released from the reference BP device. This protocol was conducted during the three arm-exercise intervention sessions, as shown in Fig. 1b. Our study employed an arm exercise to perturb BP because it was proven easier to perform and to increase subject BP value in an effortless manner. Every subject was instructed to sit relaxed to obtain the first session data (B1). Male and female subjects then performed arm exercises (E1) with 3-kg and 1-kg dumbbells (or handgrips), respectively. The next R2 to E3 followed a similar protocol. Each session was conducted for 20 min and three BP signals were collected every session and averaged for the session BP value.

### Signal processing and reference point detection

**Figure 2:**
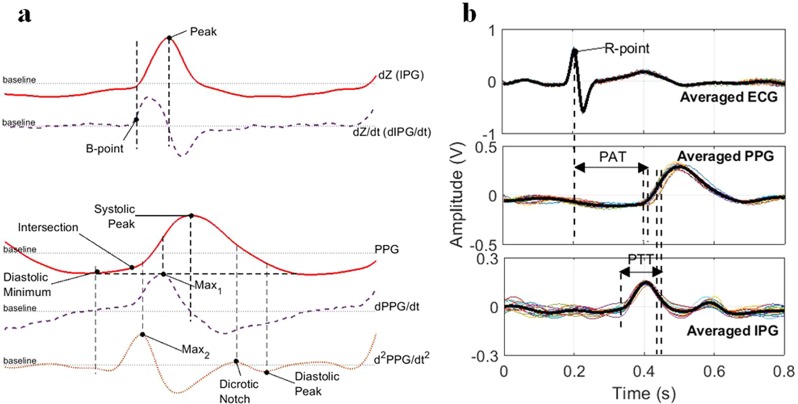
(**a**) Overall detected reference points, and (**b**) PAT and PTT calculation from three different signals.

The original sampling frequency of 500 Hz was down-sampled to 250 Hz for convenient processing. Low-frequency baseline noise in IPG and PPG signals were removed with the wavelet decomposition and moving average filter. Here, we decomposed the signal using discrete wavelet transform (DWT) with wavelet symlets 4 into 10 levels and set the approximation coefficients at the lowest frequency band to zero, as the baseline wander frequently occurred in the lower frequency band^16^. The next step was signal segmentation based on the ECG R wave and PPG systolic peak. Thus, the ensemble average of the individual pulse wave within 10 s or approximately 10–15 periodic signals was used to calculate the reference points PAT and PTT as illustrated in Fig. 2. Two reference points were detected in the IPG signal, which was the IPG peak (the maximum of the IPG (dZ) signal) and IPG B point (the location of zero crossing prior to the maximum dIPG/dt (dZ/dt)^5,17^. Moreover, seven reference points were detected from the PPG, first derivative PPG (dPPG/dt), and second derivative PPG (*d*^2^*PPG*/*dt*^2^). We determined (i) the “PPG systolic peak” from the maximum of the PPG signal, (ii) the “PPG diastolic minimum” from the valley of the PPG signal, (iii) the “PPG *max*_1_” from the maximum of the dPPG/dt, (iv) the “PPG *max*_2_” from the maximum of the *d*^2^*PPG*/*dt*^2^, (v) the “PPG intersection” from the meeting point between the line through the PPG diastolic minimum point and a tangent line from the PPG *max*_1_ point, (vi) the “PPG dicrotic notch” from the e-wave of *d*^2^*PPG*/*dt*^2^ or the small peak after *max*_2_, and (vii) the “PPG diastolic peak” from the second smaller valley after the PPG *max*_2_^16^. The overall detected reference points are shown in Fig. 2a. Therefore, we calculated the conventional PAT as the time delay between the ECG R wave and a specific point in the PPG signal (in this particular experiment we used “PPG *max*_1_”), and PTT as the time delay between the IPG and PPG signal waveform. In the case of PTT, the multiple time delay noted as *f*_1_−*f*_14_ was extracted, with the IPG signal acting as the proximal waveform and PPG signal acting as a distal waveform, which are explained by Table 1 and Fig. 2b, respectively. In addition, the radial impedance Z was obtained as follows:

1 $$Z={Z}_{b}+dZ=\frac{{R}_{cal}}{{V}_{Rcal}}({V}_{0}+\frac{{V}_{s}}{{V}_{s}^{\text{'}}}{V}_{dZ})$$

This was calculated by adding the basal impedance (Z_b_) and impedance variation (*dZ)*. Here, V_Rcal_ is the voltage drop over the calibration resistor R_cal_, V_o_ is the output voltage of the demodulation stage, V_s_ is a source sine-wave voltage, ${V}_{s}^{\text{'}}$ is the measured voltage after the final amplification, and V_dZ_ is the output voltage of the IPG waveform from the proposed system^16,18^.

**Table 1:** Different PTT feature correlation coefficient values with SBP and DBP values.

IPG	PPG	Features	*r* (SBP)	*r* (DBP)
B-Point	Max_1_	*f*_1_	0.12 ± 0.27	0.29 ± 0.23
Peak		*f*_8_	−0.48 ± 0.38	0.64 ± 0.24
B-Point	Systolic Peak	*f*_2_	0.01 ± 0.22	0.14 ± 0.18
Peak		*f*_9_	0.52 ± 0.42	−0.62 ± 0.21
B-Point	Dicrotic Notch	*f*_3_	0.25 ± 0.39	0.13 ± 0.63
Peak		*f*_10_	0.51 ± 0.32	0.44 ± 0.22
B-Point	Diastolic Minimum	*f*_4_	−0.23 ± 0.33	−0.25 ± 0.45
Peak		*f*_11_	−0.48 ± 0.30	−0.60 ± 0.30
B-Point	Intersection Tangent	*f*_5_	0.38 ± 0.25	0.31 ± 0.40
Peak		*f*_12_	−0.64 ± 0.24	−0.60 ± 0.22
B-Point	Diastolic Peak	*f*_6_	0.06 ± 0.63	0.05 ± 0.50
Peak		*f*_13_	0.06 ± 0.60	0.64 ± 0.24
B-Point	Max_2_	*f*_7_	0.46 ± 0.23	0.52 ± 0.21
Peak		*f*_14_	−0.81 ± 0.08	−0.78 ± 0.09
ECG-PAT			−0.83 ± 0.17	−0.63 ± 0.14

### BP estimation model

We tested the extracted 14 PTT features with the two types of personalized BP estimation models (i.e., six- and one-point calibration models). The six-point fitting model (PTT BP model) was in the form of an exponential function specifically as follows:

2 $$P=A.{e}^{B.PTT}$$

we first computed the correlation coefficient (*r*) between each PTT value to its corresponding BP (SBP and DBP) value. We calibrated each PTT to the reference BP level by using (2), and found the subject-specific exponential parameter of A and B. Thus, we computed the root-mean-squared-error (RMSE) between the predicted and reference BP levels per subject. Furthermore, the one-point calibration model (PTT-Z model) was obtained as follows:

3 $$P={P}_{0}+\rho \frac{{D}^{2}}{PT{T}^{2}}\,\mathrm{ln}(1+K({Z}_{max0}-{Z}_{min})$$

with subject specified constant K given as:

4 $$K=\frac{\exp (\frac{SB{P}_{0}-DB{P}_{0}}{\rho }\frac{PT{T}_{0}^{2}}{{D}^{2}})-1}{{Z}_{{\max }0}-{Z}_{{\min }0}}$$

where *ρ* is the blood density, *ρ* is defined as a constant for all subjects. Furthermore, the first calibration data were used to obtain *SBP*_0_ and *DBP*_0_ from the reference device and *PTT*_0_ and *Z*_0_ from our proposed multimodal biosensor. The data were also thus to calculate the P and K values as mentioned in (3) and (4), respectively, where P is denoted as BP (SBP and DBP) and PTT is in the range from *f*_1_-PTT to *f*_14_-PTT. Therefore, the created model (3) are used for the validation experiment to estimate the remaining data collected from our designed arm-exercise intervention experiment.

## Results

### Correlation between extracted PTT features with BP

**Figure 3:**
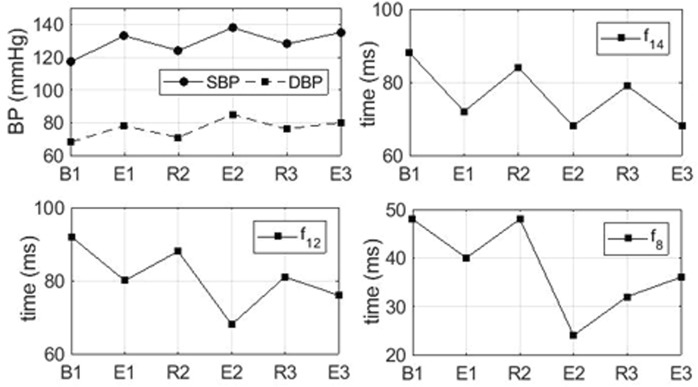
Representative example of the average SBP, DBP, f_14_-PTT, f_12_-PTT, and f_8_-PTT for each baseline and exercise session from Subject #1.

Table 1 shows the correlation coefficient *r* of the BP with 14-PTT features, averaged from all 10 subjects. As the table shows, in general, IPG B-point-based PTTs have a weaker correlation with both SBP and DBP compared to the IPG peak-point-based PTT. This is because the B point was not always clearly identifiable^18,19^, and initially, the B point was detected from the thoracic impedance using the ICG waveform^19^. However, IPG peak-based PTTs correlated well with the BP value, particularly f_12_-PTT and f_14_-PTT with −0.64 ± 0.24 and −0.81 ± 0.08 for SBP and −0.60 ± 0.22 and −0.78 ± 0.09 for DBP. Here, we found out that the f_14_-PTT shows the best correlation to both SBP and DBP. Interestingly, some features such as f_8_-PTT and f_9_-PTT have a relatively positive and medium correlation to the DBP and SBP, respectively. The positive correlation results also revealed by another study that analyzed similar subject characteristics^20,21^. Zheng *et al*.^20^ studied a cuff-less armband system using 10 young healthy subjects in the age range of 27 ± 3 years old, and PTT from five of the subjects positively correlated with the BP value during a daytime measurement. Fig. 3 shows the example of the PTT trend in different intervention sessions gathered from Subject #1.

### Comparison between extracted features with PAT

We also measured conventional PAT values in which the chest ECG R wave and wrist PPG foot were used as proximal and distal timing references, respectively. As Table 1 shows, the conventional PAT highly correlated with the SBP value (−0.83 ± 0.17) but had a relatively medium correlation to the DBP value (−0.63 ± 0.14). As previously described, f_14_-PTT extracted from the proposed system strongly correlated with both SBP and DBP. Thus, a comparison shows that PAT and f_14_-PTT both highly correlate to the SBP, where PAT was slightly better than f_14_-PTT (by 2.5%). However, our f_14_-PTT correlated better to the DBP than did the conventional PAT (by 24%).

### Performance of BP estimation model with different features

The overall performance of cuff-less BP estimation using different PTT features (f_1_–f_14_) extracted from our proposed system are shown in Table 2. Here, we computed the RMSE and *r* between the estimated SBP and DBP to the reference value from Oscar 2. The RMSE and *r* values shown in Table 2 were averaged from all subjects. Moreover, the average values of reference SBP and DBP during the measurement of all data samples were 122 ± 16 mmHg and 72 ± 8 mmHg, respectively. In addition, the best features of f_14_-PTT showed an average RMSE and *r* of 4.20 ± 1.66 and 0.79 ± 0.07 for SBP, respectively, and 2.90 ± 0.90 and 0.77 ± 0.10 for DBP, respectively.

**Table 2:** Performance of PTT-based BP estimation model with different features.

Features	SBP		DBP	
	RMSE	*r*	RMSE	*r*
*f*_1_	6.58 ± 2.04	0.30 ± 0.12	4.28 ± 1.13	0.34 ± 0.22
*f*_2_	6.69 ± 1.77	0.20 ± 0.12	4.57 ± 1.11	0.21 ± 0.12
*f*_3_	6.05 ± 2.12	0.44 ± 0.20	4.38 ± 1.16	0.37 ± 0.09
*f*_4_	6.17 ± 2.17	0.31 ± 0.29	3.79 ± 1.23	0.36 ± 0.34
*f*_5_	6.53 ± 1.86	0.27 ± 0.15	4.21 ± 1.04	0.40 ± 0.17
*f*_6_	5.68 ± 2.46	0.52 ± 0.25	4.15 ± 1.26	0.42 ± 0.23
*f*_7_	6.41 ± 2.06	0.36 ± 0.15	4.12 ± 1.33	0.46 ± 0.20
*f*_8_	5.33 ± 1.88	0.59 ± 0.22	3.19 ± 0.96	0.64 ± 0.23
*f*_9_	5.09 ± 0.56	0.56 ± 0.23	3.38 ± 0.41	0.58 ± 0.24
*f*_10_	5.19 ± 1.29	0.51 ± 0.31	3.87 ± 1.30	0.45 ± 0.27
*f*_11_	5.12 ± 0.93	0.49 ± 0.34	3.36 ± 0.72	0.52 ± 0.31
*f*_12_	4.74 ± 1.29	0.59 ± 0.29	3.07 ± 0.93	0.58 ± 0.29
*f*_13_	5.75 ± 2.45	0.51 ± 0.24	4.19 ± 1.29	0.41 ± 0.23
*f*_14_	4.20 ± 1.66	0.79 ± 0.07	2.90 ± 0.90	0.77 ± 0.10

**Figure 4:**
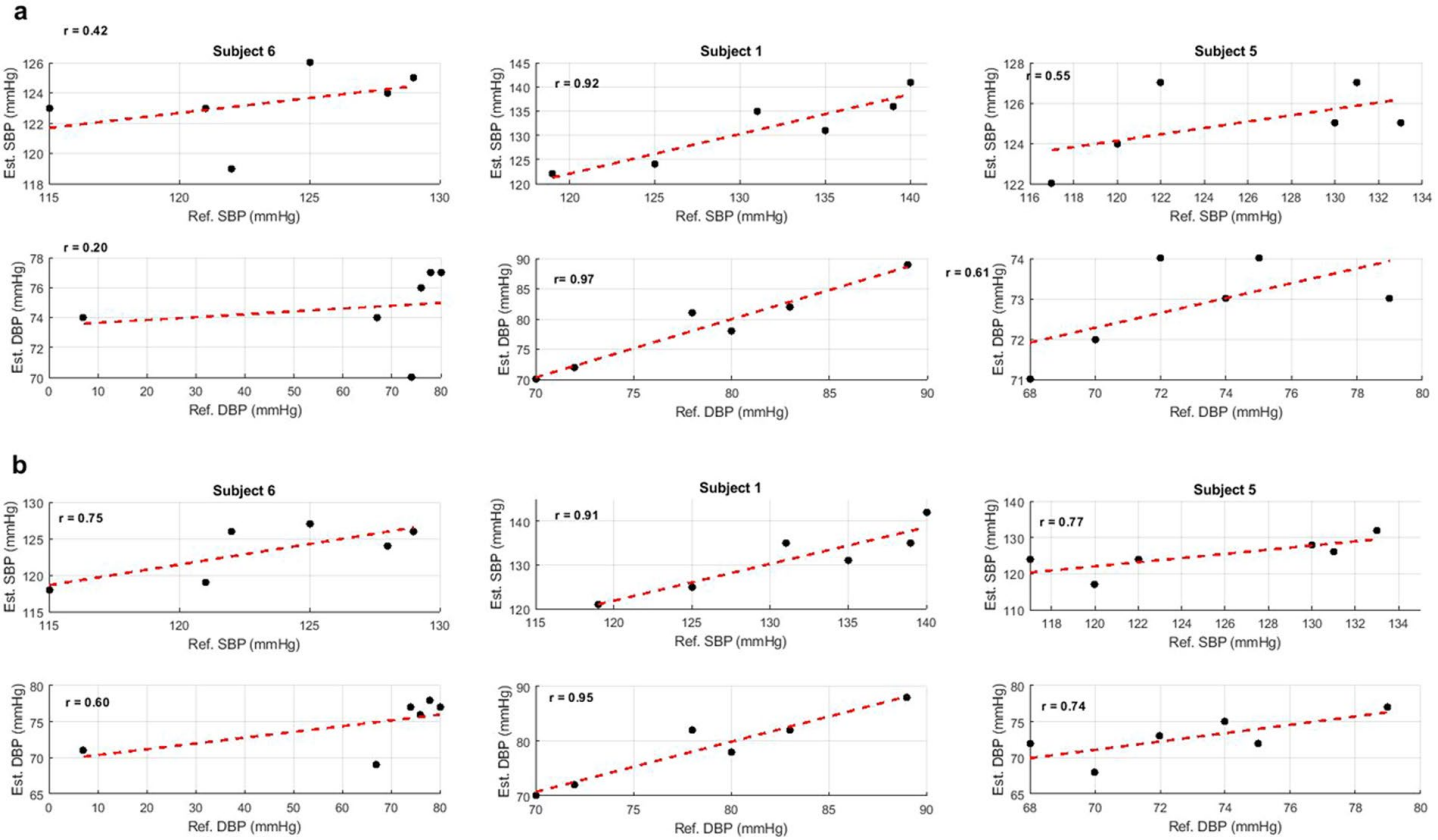
Representative estimated SBP and DBP value versus Reference value collected from Oscar 2 in three subjects. (**a**) Estimated model using input from f_12_-PTT, and (**b**) Estimated model using input from f_14_-PTT.

Fig. 4 shows the results of an exponential function fitting between f_12_-PTT (Fig. 4a) and f_14_-PTT (Fig. 4b) to the SBP and DBP in different subjects. The fitting function used for data analysis in Table 2 and Fig. 4 was defined as an exponential function in (2). This result was already proven by the previous study that showed an exponential function fits better in the plot between BP levels and PTT than other functions such as the log-natural or 1/*PTT* function^22^. Fig. 4a,b clearly show that for the representative example Subject #6, the *r* value of f_14_-PTT was superior to the f_12_-PTT in terms of both estimated SBP and DBP values. The f_12_-PTT reached only 0.42 and 0.20 for SBP and DBP, respectively, whereas the f_14_-PTT reached 0.75 and 0.60 for SBP and DBP, respectively. Example subject #5 also followed a similar trend in which f_14_-PTT was superior to f_12_-PTT. Therefore, just as the Subject #1 example yielded representative results when f_12_-PTT was strongly correlated to the BP value, so f_14_-PTT produced a similar performance.

### Comparison between PTT-based BP and PTT-Z BP models

**Figure 5:**
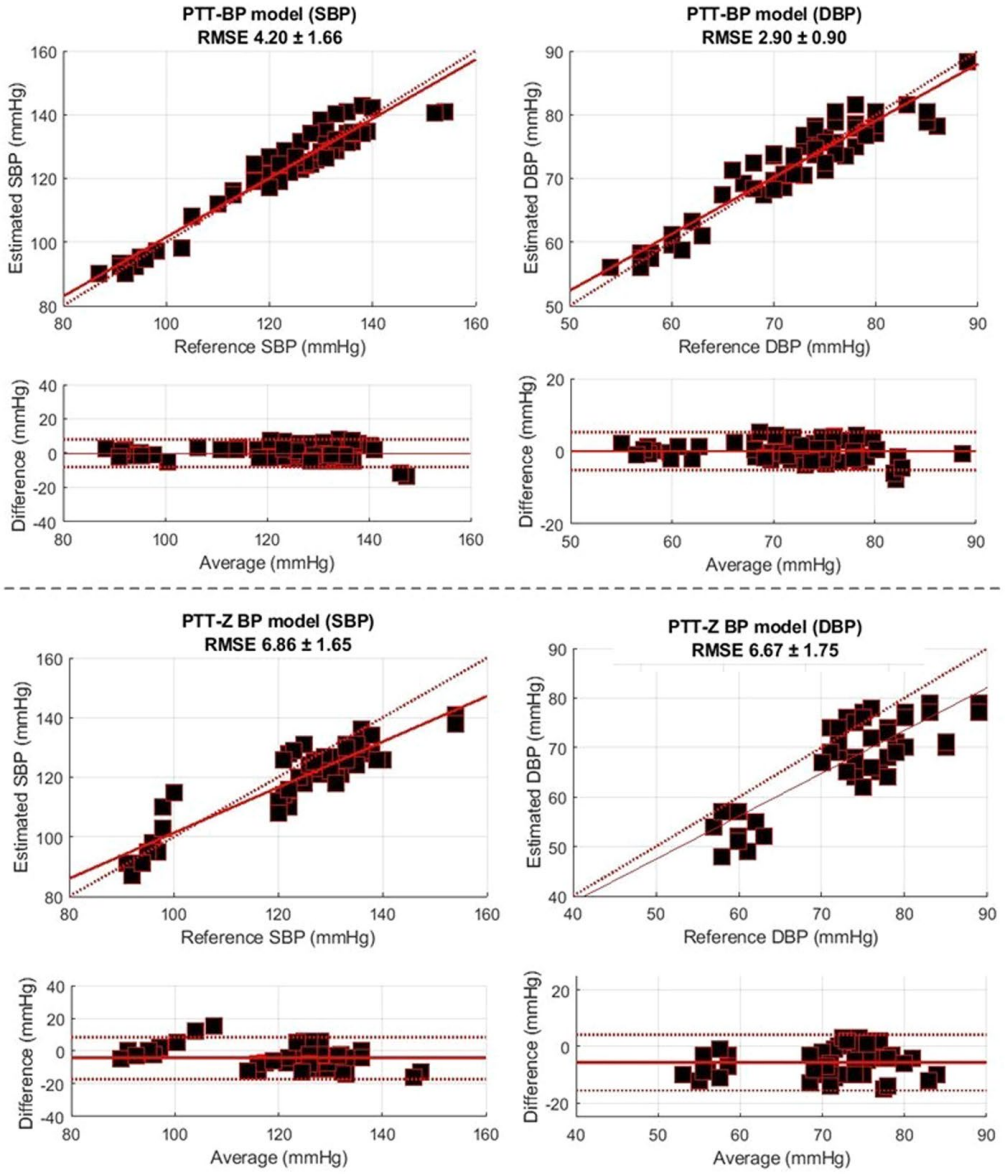
Performance comparison of the PTT-BP and PTT-Z BP models.

The Bland-Altman plots of BP associated with the PTT BP and PTT-Z BP models from all subjects are shown in Fig. 5a,b, respectively. Both figures show that we used only the feature f_14_-PTT, as it was determined from our previous analysis to be the best feature for our system. The figure shows that the RMSE from the PTT-Z BP model was higher than that of the PTT BP model with both SBP and DBP values of 6.86 ± 1.65 and 6.67 ± 1.75, respectively. One main difference between the PTT-based BP and PTT-Z BP model was the number of calibration points. For calibration, the PTT-based BP model used six points from the designed arm-exercise intervention sessions for model fitting, whereas the PTT-Z BP model^15^ used only the first point from the first B1 session. In our attempt to create a fully non-intrusive system with our cuff-less BP monitoring system, calibration was one of the biggest problems we encountered^5^. By only using one-point measurement for calibration, our system is optimal for practical implementation in personalized wearable cuff-less BP monitoring systems. However, the six-point PTT BPP model was still superior to the one-point PTT-Z model. Thus, using a greater number of calibration points is recommended to achieve more accurate results.

## Discussion

Here we calculated PTTs from the IPG and PPG signal of the wrist. Both signals have been widely known for arterial volume change detection, where PPG signal is based on the changes of reflected light due to the changes of arterial volume and dimension while IPG signal is based on the changes in the electrical impedance. The advantages of using multimodal signal is that each the signal will share a different useful further clinical information, such IPG signal may further be used for *in vivo* blood characterization including body fat analysis^23^, and body fluid monitoring^24^, while PPG signal may further be used for pulse rate variability (PRV) monitoring^25^, and mental stress monitoring^26^. With these notable aspects in mind, to achieve our final goal that develops a fully compact smartwatch-type clinical analysis system, this study proposes to employ the multimodal biosensor.

We defined 14 PTT-features from f_1_–f_14_ to identify the PTT that is most suitable for our proposed multimodal biosensor cuff-less BP monitoring system. Here the 14-PTT features were chosen carefully from the literature study of the previous works in the similar research area, the previous studies revealed that the definition of PTT differs from one researcher to another^27–30^. The most common distal waveform “foot” location is defined as the diastolic minimum time, the maximum 1^st^ derivative, the maximum 2^nd^ derivative and intersection tangent^5,27^. However, several studies have indicated that an alternative location to the “PPG foot” can also be considered, such as maximum inclination in the PPG^28,29^, systolic peak^30^, and dicrotic notch^31^. In addition, the diastolic peak analyzed in this study derives for extensional comparison data. As a result of our comparison of 14 PTT-features to the BP value, the f_14_-PTT strongly correlated to both the SBP and DBP values. As previously defined, f_14_-PTT uses PPG-foot from a maximum 2^nd^ derivative of the PPG signal as a marker from the distal waveform. Our results were similar to those of previous studies that indicated that the distal “foot” was superior to the distal “peak” as the marker for the BP estimation method^5,12^.

The IPG signal was used for the PTT calculation and radial impedance measurement. In (1), we showed how to convert the collected voltage value of IPG (*V*_*dZ*_) into the radial impedance Z. The Z value is affected by the arterial cross-sectional area (A), shown as follows:

5 $$A=\frac{\rho L}{Z}$$

where L is the length of the measured segment. Thus, the arterial cross-sectional area or arterial diameter itself proved to be highly correlated with the change in BP value^15,32^. In addition, earlier studies mentioned that the arterial cross-section has a nonlinear relationship to the arterial BP^33,34^. Therefore, we reasoned that IPG can be used as a proximal waveform and Z can be used to estimate SBP and DBP.

The results of this study show that our system is capable of using only one calibration point, which is suitable for practical application. For the one–calibration-point model, we combined the PTT and Z variables. Some researchers mentioned that for a one-calibration-point BP model, relying on only a single PTT value is difficult. Ding *et al*.^27^ combined the PTT and PPG intensity ratio (PIR) for their BP model and found that PIR could be used to improve cuff-less BP measurement. In addition, PWA and a modified normalized pulse volume (mNPV) were proven to be potential reliable markers for the BP estimation model^6,35^. In this study, we used Z, as it was evident that the power spectrum density (PSD) of Z covers both high and low components of the BP waveform. In addition, as previously explained, it is inversely related to BP.

Our study yet has limitations. Firstly, the recruited subjects of the study were rather homogenous to the young healthy adult. It is previously mentioned that the largest increases in hypertension prevalence have occurred in the young adult population, however further studies that assessed on a larger number of subjects and examining whether the PTT features and estimation model differ depending on the population, such as overweight subjects, are needed. Secondly, we did not examine the day-to-day changes related to the performance of the model, thus a further experiment needs to be conducted to test the maximum validation of the model. The proposed system is ultimately used for daily BP monitoring however this study focuses on investigating the novel non-intrusive wrist biosensor configuration for BP estimation. Thirdly, we used a commercialized LED emitter and photodiode to detect PPG signal, thus a high-efficiency, flexible and polarization insensitivity can be considered further for PPG signal detection^36^. Lastly, to realize as a 24-hour ambulatory BP monitoring system, the further experiment is required related to the system performance during mean blood pressure (MBP) monitoring and low signal-to-noise (SNR) ratio during the subject’s activity.

## Conclusion

In this study, we investigated a novel wearable BP monitoring system that utilizes multimodal wrist biosensors for a continuous, non-intrusive, and cuff-less BP monitoring system. Based on validated experiments on 10 subjects, we found that the f_14_-PTT feature that is defined as the time delay between peak IPG to the maximum 2^nd^ derivative of the PPG signal (*d*^2^*PPG*/*dt*^2^) shows a promising correlation to the BP value. In addition, it was able to estimate both SBP and DBP during the designed arm-exercise intervention sessions. We tested the system with six- and one-point calibration models that utilized PTT and a combination of PTT and the Z value, respectively. We found that both models performed adequately in practical implementations in a personalized wearable cuff-less BP monitoring system, where the six-point PTT BP model performed better than the one-point PTT-Z BP model. To the best of our knowledge, this is the first study that proposes wrist multimodal biosensor and investigates the wrist bio-signal for BP estimation for maximum wearability and convenience of the patients. Our proposed biosensor is a promising approach for a compact smartwatch-type clinical measurement system.

## Data Availability

The data that support the findings of this study can be obtained through e-mail from the corresponding author upon reasonable request.
